# Caprine and Ovine Genomic Selection—Progress and Application

**DOI:** 10.3390/ani14182659

**Published:** 2024-09-12

**Authors:** Linyun Zhang, Yixin Duan, Shengnan Zhao, Naiyi Xu, Yongju Zhao

**Affiliations:** College of Animal Science and Technology, Southwest University, Chongqing Key Laboratory of Herbivore Science, Chongqing Key Laboratory of Forage & Herbivore, Chongqing Engineering Research Center for Herbivores Resource Protection and Utilization, Chongqing Herbivore Engineering Research Center, Chongqing 400715, China; lyzhang023@163.com (L.Z.); duanyx315@163.com (Y.D.); zsnyolo@163.com (S.Z.)

**Keywords:** accuracy, genetic evaluation, genomic selection, goat, sheep

## Abstract

**Simple Summary:**

Genomic selection (GS) is defined as a marker-assisted selection method that uses high-density molecular markers covering the entire genome combined with phenotypic data to estimate genomic estimated breeding values (GEBV). This paper reviews the principles, methods, and influencing factors of GS for breeding by using phenotypic information and genetic relationships simultaneously. It also covers the research progress of GS in meat, fiber, dairy, and reproductive traits in sheep and goats. Compared to other livestock, especially cattle, sheep and goat breeding is relatively small-scale and has lagged behind. GS combined with genomic information can help promote the breeding process and development in commercial farming of sheep and goats. We highlight the potential for phenotypic information and genetic relationships to work together to drive the breeding process, providing valuable insights for implementing GS in sheep and goat breeding.

**Abstract:**

The advancement of sequencing technology and molecular breeding methods has provided technical support and assurance for accurate breeding. Genomic Selection (GS) utilizes genomic information to improve livestock breeding, and it is more accurate and more efficient than traditional selection methods. GS has been widely applied in domestic animal breeding, especially in cattle. However, there are still limited studies on the application and research of GS in sheep and goats. This paper outlines the principles, analysis methods, and influential factors of GS and elaborates on the research progress, challenges, and prospects of applying GS in sheep and goat breeding. Through the review of these aspects, this paper is expected to provide valuable references for the implementation of GS in the field of sheep and goat breeding.

## 1. Introduction

The traditional breeding methods select excellent domestic animals by estimating breeding value (EBV) based on phenotypic information and genetic relationships. The development of sequencing technology, bioinformatics analysis, and molecular breeding methods has enabled the selection index method and the best linear unbiased prediction to combine with genomic information of animals for more accurate calculation of EBV [1]. Currently, marker-assisted selection [2] and GS [1] are the major breeding techniques using genomic information of animals for breeding. Marker-assisted selection (MAS) works by adding the target trait affecting molecular markers, such as microsatellites, single nucleotide polymorphisms (SNPs), insertions and deletions, and quantitative trait loci (QTLs), to improve genetic evaluation [3,4]. Genomic Selection (GS) is a marker-assisted selection method that uses high-density molecular markers across the entire genome, combined with phenotypic data, to estimate Genomic Estimated Breeding Values (GEBVs). Compared with MAS, GS uses almost all the markers from the whole genome for EBV estimation, which provides more variation information and solves problems such as the difficulty in detecting genes or markers linked to QTL. Moreover, it greatly improves the accuracy of prediction. Many studies have been conducted on GS of livestock, and major progress has been made. Currently, GS has been widely applied in the livestock industry, particularly in dairy cattle and pigs. It has become a routine breeding method in dairy cattle rapidly [5] and has achieved significant improvement in the accuracy of predictions compared to traditional methods. However, the research on GS in sheep and goats is not yet complete and requires further investigation. At present, the sheep and goat industry faces the bottleneck of slow growth and low reproductive efficiency. Compared to other livestock, the foundation of sheep and goat breeding is relatively weak, with poor breeding infrastructure, outdated breeding methods, and inadequate fundamentals, such as performance testing and genetic evaluation. Therefore, it is necessary to continue enhancing the capacity for innovative breeding methods. Improvements in the speed and accuracy of genome sequence, as well as reductions in cost, mean that GS is expected to become a routine method in sheep and goat breeding. GS has become a routine method in cattle breeding, with official genomic evaluation platforms established in many countries and regions for regular genomic evaluation [6]. With the advancement and improvement of molecular breeding technology and the reference genomes of sheep and goats, GS has become more prevalent in their breeding programs. This will further drive the progress of genetic breeding for sheep and goats. This review provides a comprehensive overview of the principles, methodologies, and influential factors of GS, focusing on its application in sheep and goat breeding. By summarizing recent research and discussing the challenges and future prospects, the article aims to offer valuable insights for effectively implementing GS to enhance traits such as meat, fiber, dairy, and reproduction in sheep and goats.

## 2. Methodology

This review utilizes a literature review methodology to investigate the principles, methods, and factors affecting GS in sheep and goat breeding. A structured and systematic approach is adopted, involving a comprehensive analysis of secondary data sourced from published literature, scientific reports, and relevant databases. The specific steps of this process are detailed below.

### 2.1. Data Collection

Sources: The study utilizes peer-reviewed journal articles, books, industry reports, and credible online sources. Databases: PubMed, Web of Science, China National Knowledge Infrastructure, and Elsevier are used to gather relevant literature. Key search terms are listed in Table 1.

### 2.2. Selection Criteria

The inclusion criteria are as follows: publications from the last 20 years; studies focusing on sheep and goats; peer-reviewed articles, research papers, review articles, and meta-analyses.

The exclusion criteria are as follows: articles with insufficient empirical data (for example, studies with a sample size of fewer than 10 cases, articles that do not provide a clear and detailed description of the experimental methods, etc.); studies not directly related to the research questions (for example, studies whose topics are not directly related to genomic selection (GS) in sheep and goats, research that primarily focuses on policy, economic analysis, or social sciences rather than biology or genetics, etc.)

### 2.3. Selection Process

(1) Title and abstract screening for relevance;
(2) Full-text review for inclusion based on criteria;
(3) Final selection of 62 highly relevant articles.

### 2.4. Data Extraction and Analysis

Data from the selected articles were extracted using a standardized form. The following information was recorded: study objective; species (sheep or goat); traits analyzed (meat, fiber, dairy, reproductive); GS methodologies used; key findings and conclusions. The extracted data were then analyzed to identify common themes, methodologies, and conclusions, which formed the basis of the review presented in this paper.

## 3. Genomic Selection (GS)

### 3.1. The Principle of GS

**Figure 1:**
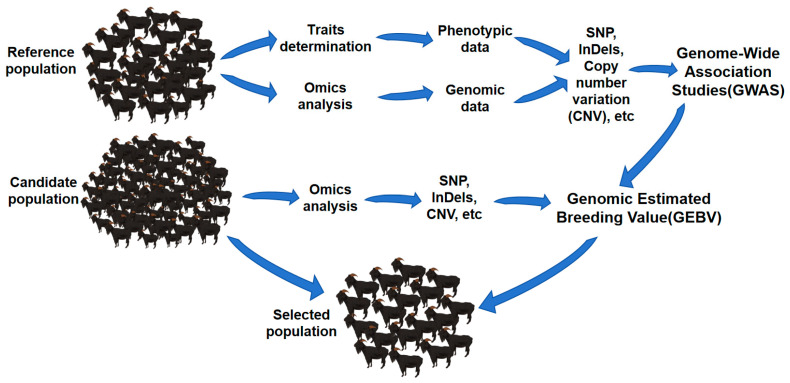
GS technical route.

GS, which uses whole-genome information to select superior livestock, was first proposed in 2001 by Meuwissen [1]. GS estimates the effect value of each molecular marker or chromosome segment by using the whole-genome marker information and phenotype information of individuals. By integrating all effect values, the EBV of an individual with genomic information, also known as the GEBV, could be obtained. The specific procedure of GS includes the following: first, a reference population with both genotype and phenotype information needs to be constructed; then, the genotype information is used to obtain whole-genome marker information; to find the best calculation model, the effective value of each marker was calculated by different models; next, the best model is used to calculate the individual GEBV of the candidate population based on its genomic information; finally, individuals with higher GEBV are selected. The technical route of GS is shown in Figure 1.

Compared to other breeding methods, GS, which uses genomic information, is less dependent on phenotypic information, so it can use the genomic information of candidate populations for early selection to shorten the generation interval [7], and it is beneficial for the selection of limiting traits and low-heritability traits, further reducing breeding costs to a certain extent. GS assumes that there is linkage disequilibrium (LD) between the quantitative trait loci (QTLs) and at least one molecular marker, then identifies all QTLs that affect the target trait to achieve more accurate predictions of individual breeding values. Selection of candidate individuals by estimating GEBV can distinguish the differences among individuals and improve the intensity of selection [8]. GS is based on high-throughput sequencing technologies, such as SNP chips, whole-genome sequencing, and simplified genome sequencing, among others. With the development of sequencing technology, the accuracy of whole-genome sequencing and SNP chips has improved, and the cost has gradually decreased, leading to wider applications in breeding.

### 3.2. Methods for GEBV Estimation

The essence of GS lies in the estimation of genomic breeding value, which is commonly achieved through the methods of Best Linear Unbiased Prediction (BLUP) and Bayesian inference. BLUP assumes that all markers are micro-effect markers, while all markers conform to a normal distribution with the same variance. The traditional BLUP uses the relatedness matrix (A) to predict the EBV of an individual, and methods using other relationship matrices for estimation have emerged based on BLUP. Ridge Regression Best Linear Unbiased Prediction (RRBLUP) includes individuals as random effects and calculates marker effects using matrix A, which is constructed from the genetic information of both the reference and predicted populations. Genomic Best Linear Unbiased Prediction (GBLUP) replaces relatedness (matrix A) with genomic marker information (matrix G) and uses both genomic marker information and phenotypic information of the reference population to estimate the individual GEBV of the candidate group. Based on GBLUP, single-step GBLUP (ssGBLUP) uses the integrated information of matrix A and matrix G to construct matrix H; it directly estimates the GEBV of individuals in the candidate group within the framework of the mixed model system of equations.

BLUP is a linear model in nature, and the mixed linear model is mostly used to predict the individual breeding value. The commonly used mixed linear model is written as follows:

(1) y=Xb+Za+e

In this formula, *y* is the vector of observations, *b* is the vector of fixed effects of all fixed factors, *a* is the vector of random effects of all random factors, *X* and *Z* are the structure matrices of *b* and *a*, respectively, and *e* is the vector of random residual effects. MME (mixed model equations) is a useful computing algorithm for obtaining BLUP; the matrix A used by BLUP is the kinship matrix (additive correlation matrix between individuals), and the MME of BLUP is as follows:

(2) $$\left[\begin{array}{cc} X^{\prime }X & X^{\prime }Z\\ Z^{\prime }X & Z^{\prime }Z+A^{-1}k \end{array}\right]\left[\begin{array}{c} \hat{b}\\ \hat{a} \end{array}\right]=\left[\begin{array}{c} X^{\prime }y\\ Z^{\prime }y \end{array}\right],k=\frac{\sigma_{e}^{2}}{\sigma_{a}^{2}}$$

On the basis of BLUP, more studies have used the genomic data of animals to predict the EBV of individuals more accurately and quickly, and the EBV obtained by using genomic information for prediction is GEBV. SNPBLUP (RRBLUP), which assumes that all markers have an effect, uses the BLUP model to estimate the SNP effect, which sums the effects of SNPs spread across the genome. GEBV is indirectly calculated by estimating the marker effects, where GEBV is the sum of the marker effects, and the linear model is as follows:

(3) $$y=Xb+\sum_{j=1}^{m}M_{j}g_{j}+e$$

In this formula, *y* is the vector of phenotypic values for individuals of the reference group, *g_j_* is the effect of the *j*-th marker, *M_j_* is the genotype vector of the *j*-th marker, and m denotes the number of markers. The MME of SNPBLUP is as follows:

(4) $$\left[\begin{array}{cc} X^{\prime }X & X^{\prime }M\\ M^{\prime }X & M^{\prime }M+Ik \end{array}\right]\left[\begin{array}{c} \hat{b}\\ \hat{g} \end{array}\right]=\left[\begin{array}{c} X^{\prime }y\\ M^{\prime }y \end{array}\right],k=\frac{\sigma_{e}^{2}}{\sigma_{a}^{2}}$$

With the development of sequencing technology, selection methods using whole genome data have advanced. The genomic matrix G is constructed using whole genome information to predict GEBV by GBLUP, and the linear model is as follows:

(5) y=Xg+Za+e

In this formula, *y* is the vector of observations, *g* is the genotype effect, *a* is the additive effect, *e* is the residual vector, *X* is the matrix of observations of genotypes at a single locus, and Z is the matrix of additive effects. GBLUP uses genome matrix G (realized relationship matrix) to predict GEBV, and its MME is as follows:

(6) $$\left[\begin{array}{cc} X^{\prime }X & X\prime Z\\ Z\prime X & Z^{\prime }Z+A^{-1}k \end{array}\right]\left[\begin{array}{c} \hat{b}\\ \hat{a} \end{array}\right]=\left[\begin{array}{c} X^{\prime }y\\ Z^{\prime }y \end{array}\right],k=\frac{\sigma_{e}^{2}}{\sigma_{a}^{2}}$$

The BLUP prediction method that uses genome matrix G combined with the kinship matrix A to establish the mixing matrix H is ssGBLUP, and its linear model is as follows:

(7) $$y=Xb+Z_{i}a_{i}+e$$

In this formula, *y* represents the vector of observations, *b* represents the vector of fixed effects, *a_i_* represents the vector of estimated breeding values, *e* represents the residual vectors, and *X* and *Z* are the design matrices for *b* and *a*, respectively. Its MME is as follows:

(8) $$\left[\begin{array}{cc} X^{\prime }X & X^{\prime }Z\\ Z^{\prime }X & Z^{\prime }Z+H^{-1}k \end{array}\right]\left[\begin{array}{c} \hat{b}\\ \hat{a} \end{array}\right]=\left[\begin{array}{c} X^{\prime }y\\ Z^{\prime }y \end{array}\right]\;k=\frac{\sigma_{e}^{2}}{\sigma_{a}^{2}}$$

The principle of the Bayesian method is to use the prior distribution and the actual observations to form the posterior information, and then perform statistical inference on the posterior information. Based on the variances of the markers, Bayesian methods are divided into Bayes A, Bayes B, Bayes Cπ, Bayes R, and so on [9]. Bayes A assumes that the SNP effect distribution conforms to a normal distribution and that each SNP effect is specific. Bayes B assumes, on the basis of Bayes A, that most SNPS have no effect, and only a few SNPs have an effect, with these effects being specific. Bayes Cπ sets all nonzero SNP effects to be equal, based on Bayes A and Bayes B. Bayes R assumes that each normal distribution is different and that the labeled effects follow a mixture of normal distributions. The basic model of the Bayesian method is as follows:

(9) $$y_{i}=\mu +\sum_{j=1}^{n}\delta_{j}x_{ij}g_{j}+e_{i}$$

In this formula, *y* represents the observation vector, *x_ij_* represents the SNP genotype, *g_j_* represents the SNP effect, and *j* is the SNP effect indicator variable. The GEBV value of the sample is as follows:

(10) $${GEBV}_{i}=\sum_{j=1}^{n}x_{ij}{\hat{g}}_{j}$$

Using Bayesian methods is more difficult and time-consuming than BLUP. At present, GBLUP and ssGBULP are commonly used to estimate the GEBV of individuals.

### 3.3. Influential Factors of the GS Accuracy

Although GS has certain advantages in various breeding methods, its accuracy is also affected by some factors. The main influencing factors are shown in Table 2, and the specific influencing mechanisms are as follows:

Size of the reference population. The size of the reference population has a great impact on the accuracy of GS. As the size of the reference population increases, the accuracy of the GEBV for individuals in the candidate population improves [10]. For example, in the GS of goats, Carillier [11] constructed different levels of reference populations (20,705 bucks and 1946 does) for estimating the GEBV using Alpine dairy goats and Saanen dairy goats, respectively, in order to resolve the reference population size issue. The results showed that the accuracy of individual GEBV of the candidate population increased from 2% to 31% with the increase in reference population size. Moreover, the structure of the reference population also has a certain impact on the accuracy of GEBV. Raoul [12] explored the influence of reference populations with different population structures on the accuracy of GEBV and found that different sex ratio structures of reference populations had a certain influence on GEBV. Wei [13] investigated the effect of different population sizes on the accuracy of GS in Chinese pigs and found that genome prediction accuracy was positively correlated with the size of the reference population. Meanwhile, some studies have shown that the accuracy of EBV prediction tends to be stable when the reference population reaches a certain size, which can effectively ensure the accuracy of genome selection [14].

Marker density and the degree of LD between markers. Marker density affects the accuracy of GS to a large extent. Increasing marker density can improve the degree of LD between markers. Therefore, the higher the marker density, the greater the accuracy of GS and the better the selection effect. In addition, some studies have shown that grouping SNPs based on regional LD can effectively reduce the adverse effect of the uneven distribution of LD on the genome, thereby greatly improving the efficiency of high-density SNP data for genome prediction and heritability estimation [15].

Generation intervals. With the increase in the generation interval between the reference population and the candidate population, LD between markers and QTLs can change. The shorter the generation interval and the stronger the correlation between individuals in the reference population and the candidate population, the higher the accuracy of GS. After 3–4 generations, it is necessary to re-estimate the effect of markers using a new or improved reference population [9].

Heritability of the target traits. Compared with traditional breeding methods, GS is more effective in selecting traits with low heritability. However, previous studies have shown that the heritability of target traits also affects the accuracy of GS. The greater the heritability of target traits, the higher the accuracy of GS [16]. To explore the impact of trait heritability on the effectiveness of GS, Zheng [17] used different models to conduct GS on traits with different heritability (h^2^ = 0.1, 0.3, 0.5) in cattle. The results showed that the selection effect of traits with high heritability was greater than that of traits with low heritability under different models and conditions.

Methods and models for estimating GEBV. As mentioned above, the estimation methods of GEBV mainly include BLUP and Bayes. Different traits have their own applicable models, and the suitability of models can directly affect the accuracy of GS. Baloche [18] compared the accuracy of three different models (ssGBLUP, pseudo-BLUP, and pseudo-ssGBLUP) using SNP chips and a reference population of 2892 Lacaune sheep. The results found that the accuracy of GS in each model increased by 0.1–0.2 compared with that of their parents, and conventional ssGBLUP had the highest accuracy in this population. Yan [19] conducted a simulated study of GS on body weight and wool fiber diameter in Inner Mongolia Cashmere goats, and GBLUP, ssGBLUP, Bayes A, Bayes B, Bayesian R, and Bayesian LASSO methods were used to calculate the GEBV, respectively. The results showed that the most suitable model needs to be selected in combination with the current conditions, and the most suitable model enables GEBV with the highest accuracy.

## 4. Research Progress of GS in Sheep and Goat Breeding

Breeding is an important aspect of animal husbandry. GS has good applications in livestock breeding, especially in cattle breeding [5]. Although the studies of GS on the economic traits in sheep and goats are still in a developmental stage, there has been much attention paid to it, and the platform and the system of GS study have been gradually increasing.

### 4.1. Research Progress of GS in Meat Traits of Sheep and Goats

The improvement of meat quality is conducive to the development of the sheep and goat industry. More GS studies were conducted on meat quality traits in sheep and goats than on other traits. Werf [20] conducted a simulation study on GS using a selection index method for three carcass traits of Merino sheep, which included growth rate, fat, and muscle conformation. The results showed that the application of GS in Merino sheep breeding could increase the mutton yield index by 32%, further promoting the genetic research of sheep. Daetwyler [21] estimated the GEBV for weight, loin-eye area, and fat traits in Australian sheep using a reference population of 7180 multi-breed sheep with phenotypic records (Merino sheep, etc.) and found that the accuracy of prediction for each trait ranged from 0.07 to 0.57. At the same time, the accuracy of prediction was improved by increasing the reference population. Slack-Smith [22] used the Bayes B to perform GS on three traits (carcass weight, loin-eye area, and intramuscular fat) of meat-purpose sheep and goats, and the prediction accuracy of GEBV for the three traits was 0.45, 0.72, and 0.32, respectively. Based on the Annual Genetic Gain (AGG) of the meat sheep breeding program, Shumbusho [23] optimized the GS strategy for mutton, milk, and maternal traits. The accuracy of genome prediction for meat sheep, dairy sheep, and dairy goats in a population of 2000 individuals improved by 17.9%, 51.7%, and 26.2%, respectively, compared with the traditional breeding methods. Moghaddar [24] used a multi-species reference population to estimate GEBV for 26 traits, including muscle, weight, fat, wool traits, and so on, in Australian sheep. The results showed that the accuracy of GEBV for body weight was 0.11–0.27 for Poll Dorset and White Suffolk sheep and 0.25–0.63 for Border Leicester and Merino sheep. Brito [25] used a 15K SNP chip to estimate GEBV for many traits, such as carcass traits and mutton quality traits, in New Zealand sheep from a large reference population of 14,845 individuals. The results showed that the accuracy of prediction of GEBV for carcass traits and mutton quality traits were 0.28 ± 0.09–0.55 ± 0.05 and 0.21 ± 0.07–0.36 ± 0.08, respectively. Lillehammer [26] conducted GS simulation studies under different models for carcass traits, growth traits, and maternal traits of Norwegian white sheep and found that GS increased the genetic benefits of these traits by 18% to 20%. Scholtens [27] used Bayes C to estimate the GEBV for milk yield, protein content, and somatic score of New Zealand dairy goats, and the average prediction accuracy was 0.34 to 0.43, which was higher than the traditional BLUP. Ashraf [28] used GBLUP, Bayes R, Bayes A, Bayes B, and Bayes L to perform GS and compared eight different traits of Soay Sheep, such as body weight, length of front and rear legs, coat color, patterns, and so on. The results showed that the prediction models based on the genome had high accuracy and strong correlation with each other. Combined with pedigree analysis, Hunter [29] performed genomic prediction of adult body weight in Soay Sheep and found that using genome prediction to study the microevolution of wild populations can eliminate the need for pedigree data, which is expected to open up new research systems. Oliveira [30] used ssGBLUP to predicted GEBV for the traits of birth weight, weaning weight, carcass weight, and fat in Norwegian white sheep and New Zealand sheep. The results showed that the accuracy of the GEBV was 0.13–0.44 in Norwegian white sheep, 0.06–0.15 in New Zealand sheep, and 0.10–0.41 in the mixed population of both breeds. Moghaddar [31] performed genomic prediction of body weight and mutton quality traits in Duper sheep using GBLUP, and the accuracy of genomic prediction for different traits ranged from 20.0 to 30.50. Massender [32] predicted the GEBV for eight traits in 5158 Alpine dairy goats and 2342 Saanen dairy goats, and the results showed that the theoretical accuracy of GEBV for all eight traits increased by 32–37% and 40–41% on average compared with traditional genetic evaluation, and the accuracy of GEBV estimation for multiple breeds was significantly higher than that for single breeds. With the in-depth development of GS, more studies are utilizing multi-breed approaches to further estimate GEBV for meat quality traits in sheep and goats. These GS studies using multi-breed approaches for analysis have demonstrated improved accuracy compared to single-breed approaches. The utilization of multi-breed joint predictions may potentially serve as a pathway for further enhancing the accuracy of GS in the future.

### 4.2. Research Progress of GS in Fiber Traits of Sheep and Goats

Wool, as one of the most important raw materials in the textile industry, is also one of the first natural fibers to be used. The quality of wool has a significant impact on the industry of sheep and goats and the textile industry and is affected by the length, fineness, strength, crimp, and so on [33]. At present, some progress has been made in the research of GS on wool. Daetwyler [21] used a reference population of 7180 multi-breed sheep with phenotypic records (Merino sheep, etc.) to estimate GEBV for many traits, such as wool weight, fiber diameter, and staple fiber strength, in Australian sheep. The results showed that the prediction accuracy of GBLUP, which is a representative method of GS, was slightly higher than that of BayesA for wool traits of sheep, and the prediction accuracy of GS in estimating the GEBV for Merino sheep wool traits was 0.15–0.79. Moghaddar [24] used a multi-breed reference population to estimate the GEBV for the quantity of wool in Australia by GBLUP and found that the average accuracy of the GEBV for the quantity of wool was high, between 0.33 and 0.75. Dodds [34] applied GS to a reference population of 13,364 sheep of different breeds (Romney, Coperworth, and Pollendale) and found that the accuracy of genomic prediction improved to 0.26. Bolormaa [35] used Bayes R and GBLUP to estimate the GEBV for wool quality, yield, and turbid degree in 5726 Merino sheep and crossbreed Merino sheep and found that the average accuracy of genomic prediction of wool traits was about 0.22. Moghaddar [36] used GBLUP and Bayes RC model to estimate GEBV for eight economic traits related to meat quality and wool production in Australian sheep. In purebred and crossbred populations, the average genomic estimated breeding values of GBLUP for each trait were 0.083 and 0.073, while those of Bayes RC were 0.102 and 0.087. Wei [37] used PBLUP and ssGBLUP to estimate the EBV and GEBV for wool traits in Merino sheep, and the results showed that the average accuracy of GEBV increased by 3.016–11.736% compared with EBV. Wang [38] used a reference population of 1920 individuals to estimate the GEBV for villus yield, villus length, and villus fine traits of Inner Mongolia Cashmere goats and found the prediction accuracy of villus yield, villus length, and villus fine was about 0.78, 0.79–0.80, and 0.55–0.56, respectively. The prediction accuracy of hair length was about 0.78–0.82, and the prediction accuracy of different models followed the trend of ABLUP < GBLUP < ssGBLUP, indicating that ssGBLUP has higher accuracy and advantage. Zhu [39] used a reference population of 11,500 Merino sheep from seven groups and used GBLUP to estimate GEBV for six traits, including hair length, net hair weight, average coarse fiber density, coefficient of variation of average coarse fiber density, short-fiber strength, and wool elongation. The prediction accuracy of the six traits ranged from 0.28 to 0.60. Yan [19] simulated a GS study on the wool fiber diameter of Inner Mongolia Cashmere goats and found that GS was more accurate than conventional selection methods. Those studies on GS of wool traits have shown that the accuracy of GS is higher than that of traditional selection methods, and the accuracy of GBLUP in GS wool traits of sheep and goats is higher than that of Bayes A. Studies in Cashmere goats also showed that ssGBLUP, which combines phenotypic, genealogical, and genomic information, is more accurate than GBLUP [21,24]. Moreover, in GS studies on wool traits of sheep and goats, multi-breed reference populations have been established for selection.

### 4.3. Research Progress of GS in Dairy Traits of Sheep and Goats

Goat and sheep milk contains a variety of elements required by the human body, has a nutrient ratio that is more suitable for human absorption, is easier to digest than cow’s milk, and is hypoallergenic [40]. Dairy trait is also an important economic trait of sheep and goats, and GS research for dairy traits, such as milk yield, milk protein quantity and percentage, and milk fat quantity and percentage, has also been studied to some extent. Duchemin [41] used different methods such as BLUP, Bayes Cπ, partial least squares (PLS), and sparse PLS to estimate GEBV and compare their accuracies for 14 milk traits, such as milk yield, fat content, and somatic cell score, in sheep. Duchemin’s study found that there was no significant difference in the accuracy of prediction among different methods using genomic information, and the accuracy of genomic estimation was improved by 0.4–0.6 compared to conventional BLUP. Carillier [42] performed a genomic assessment of milk production, somatic cell count, and udder traits in Alpine and Saanen dairy goats using an SNP chip, with accuracy ranging from 36% to 53%. Carillier [11] used ssGBLUP to estimate GEBV for udder traits in 677 French Alpine and Saanen dairy goats; resulting in an improvement in selection accuracy from 22% to 37%. GS. Mucha [43] used a 50k SNP chip to estimate the GEBV of 14,453 hybrid dairy goats, including British Togenberg goats, Alpine dairy goats, and Saanen dairy goats, using SNPBLUP and ssGBLUP models to estimate their GEBV for traits such as milk yield, lactation duration, and other lactation traits. The results showed that ssGBLUP was more accurate in this population, and the accuracy of GEBV estimated by ssGBLUP was 0.61. Antonio [44] used BLUP and ssGBLUP separately to estimate the EBV and GEBV of 50,649 Florida goats for dairy traits such as milk yield and found that the reliability of ssGBLUP increased by 1.06% relative to BLUP. Teissier [45] used the 50k SNP chip to estimate the GEBV for protein content in the milk of 2955 French dairy goats using ssGBLUP. The results showed that the accuracy of the prediction of ssGBLUP using genomic information increased by 5–7% compared to the traditional prediction method. The accuracy of three WssGBLUP was 6%, higher than that of the unweighted ssGBLUP. Cesarani [46] used BLUP, GBLUP, and ssGBLUP to estimate EBV and GEBV for milk fatty acid content in sheep. The study showed that the accuracy of prediction using genomic information was higher than that using traditional EBV estimation methods, and the accuracy of prediction was higher in the older group. Teissier [47] used ssGBLUP and weighted ssGBLUP (WssGBLUP) of haplotypes to perform genomic prediction for five milk production traits, five udder shape traits, and somatic score in French dairy goats and found that haplotype-based models could improve the accuracy of genomic prediction of some traits. Sousa [48] investigated the optimal GS model for milk production and composition traits in Brazilian Saanen dairy goats. Using GBLUP, Bayes Cπ, and Bayesian LASSO for estimated GEBV in the reference population, it was found that the accuracy of the three methods was consistent, while GBLUP had better selection performance and lower cost in this reference population. Massender [32] used ssGBLUP to perform GS for protein and fat content in milk from 1707 Canadian Alpine and Saanen dairy goats, and the accuracy of GEBV increased by 12–16% compared to traditional selection accuracy. Marina [49] used a low-density SNP chip to estimate EBV for milk coagulation properties and cheese yield-related traits in 2020 dairy goats using the models of BLUP, SNPBLUP, and ssGBLUP. The results showed that the prediction accuracy of ssGBLUP was improved by 0.19–0.44 after considering genomic information compared to BLUP. In the current studies on the GS for dairy traits of sheep and goats, several studies have shown that ssGBLUP has relatively superior prediction accuracy in groups with different numbers and structures [9,46,49].

### 4.4. Research Progress of GS in Reproductive Traits of Sheep and Goats

Reproductive traits are very important economic traits in sheep and goats, and their performance directly affects the development of the sheep and goat industry. Most reproductive traits have low heritability and are difficult to assess; however, GS can improve the selection efficiency for traits with low heritability. Pickering [50] performed GS on 4237 Romney sheep and estimated the GEBV for reproductive traits. The results showed that the prediction accuracy of GEBV for reproductive traits ranged from 0.16 to 0.52. The application of GS shortened the generation interval, and the estimated genetic gain had the potential to increase by up to 84%. Granleese [51] simulated GS in sheep under optimal contribution selection and found that the MOET and MOET+JIVET programs increased the genetic gain by 38–76% and 51–81%, respectively. Newton [52] used a genomic information-based simulation to model the impact of different age structures of sheep populations on reproductive performance in Australian sheep. The results showed that the availability of ram and ewe ages as well as male genomic information significantly affected the predicted genetic gain, and genetic progress was unlikely to be adversely affected at fertility rates of 10% or more. Bolormaa [53] used GBLUP to conduct GS on reproductive traits such as lambing number, litter size, and weaning weight in 5340 Merino sheep, resulting in an average accuracy of 43.0 based on parent–offspring trait deviations and 17.0–61.0 for random cross-validation of GEBV. The highest accuracy of prediction based on lambing number and litter size among the reproductive traits studied was also obtained to be between 41.0 and 54.0. Moghaddar [54] used GBLUP and Bayes R to estimate GEBV for reproductive traits in Australian Merino sheep and showed that the accuracy of genomic prediction increased by 1.0–12.0% in populations with distant genetic relationships, with an average improvement of 5.2%. The two models had similar predictive performance. This was done to improve the accuracy of genomic prediction for reproductive traits in Australian Merino sheep. Lillehammer [26] conducted a simulation experiment on maternal traits of Norwegian White Sheep under different GS models. The results showed that the implementation of GS increased the genetic gain of maternal traits in Norwegian White Sheep by 65–77%. Araujo [55] used ssGBLUP based on SNPs and haplotypes to estimate GEBV for some traits, such as birth weight, weaning weight, adult weight, and litter size, in Lacaune sheep. Araujo found that using genomic information provided similar or higher levels of predictive and theoretical accuracy compared to traditional methods across all traits and reduced the dispersion of GEBV for Lacaune sheep reproductive traits. Due to the complexity of reproductive traits, there is currently relatively limited research on GS of sheep and goat reproductive traits, and further in-depth research is needed. Compared with other traits, there are relatively few studies on the reproductive traits of GS in sheep and goats, which may be due to the low heritability of the reproductive traits.

### 4.5. Research Progress of GS in Other Economic Traits of Sheep and Goats

In addition to the above traits, GS has also been used in studies of other traits in sheep and goats. Phua [56] conducted GS research on New Zealand sheep and dairy industry to reduce the impact of facial eczema (Pithomycotoxicosis), and the best genome prediction accuracy obtained from the study was 0.38. Rowe [57] performed a GS study on enteric methane emissions in 1872 New Zealand sheep with GBLUP, and the genomic prediction accuracy was 0.37. Duijvesteijn [58] conducted a prediction of horn type in Merino sheep using traditional pedigree estimation and genomic information. The accuracy of prediction of horn type was 0.323 for females and 0.725 for males using pedigree, and 0.713 for females and 0.620 for males using genomic information. In order to increase the genetic resistance of sheep to gastrointestinal nematode infection, Dos [59] evaluated the genetic resistance using both traditional evaluation methods and GS. The results showed that genomic models provided more accurate estimates of EBV compared to pedigree-based models. Carracelas [60] used ssGBLUP to obtain GEBV for increasing the genetic resistance of sheep to gastrointestinal nematodes and selected relevant genomic regions associated with resistance in the Coopworth breed.

## 5. Discussion

The application of GS in sheep and goat breeding still needs further development. Compared to other livestock, sheep and goat breeding has a relatively small scale, and integrating GS into sheep and goat breeding also faces some difficulties and challenges, such as the reduction of accuracy due to long-term selection, incomplete data, and the lack of a large and excellent reference population. It is necessary to gain a deep understanding of the potential factors and regularly update the prediction models. In addition, recent studies have also shown an increasing trend in conducting GS research on sheep and goats through the joint analysis of multiple breeds, as several beneficial factors have been identified (Table 3). Multiple studies have indicated that utilizing joint GS analysis with multiple breeds significantly improves the accuracy of GEBV compared to studies with a single breed. The substantial number of multi-breed GS studies conducted for both meat and fiber traits of sheep and goats shows clear evidence of such a trend. Particularly, it was observed that the improvement in GEBV estimation by incorporating multiple breeds is more significant compared to single-breed GS for meat quality traits in sheep and goats [24,30,61]. This finding holds significant implications for future GS research, suggesting that combining multiple breeds for the same trait in GS analysis could potentially elevate the accuracy of GS to a higher level, especially for meat quality traits. Furthermore, the enhancement of precision for GS by combining multiple breeds for the same trait shows promising and exciting prospects for advancing selective breeding programs in the sheep and goat industry.

In the context of diverse population structures and calculation methods, the accuracy of GS may vary considerably. Table 3 presents an overview of the studies focused on the selection of phenotypic traits in sheep and goats, using multi-breed or diverse methods. These selection methods, which use genomic information, show little difference in many studies, but for those studies comparing different selection methods, ssGBLUP shows superior prediction compared with other methods. In some studies, the prediction accuracy of WssGBLUP is higher than that of ssGBLUP, thereby providing valuable guidance for prospective GS studies in sheep and goats. Furthermore, it is noteworthy that gender ratios and group structures have also been identified as potential factors influencing the accuracy of GS predictions. This insight opens up new avenues of exploration for future GS studies in this field. As such, understanding and considering these various factors can significantly contribute to the refinement of genomic selection techniques for sheep and goats.

## 6. Conclusions

GS, as it becomes a popular choice to estimate GEBV, can also obtain benefits from other interdisciplinary developments, such as the advancement of data analysis tools, the development of machine learning and deep learning for optimizing models, gene editing, transgenics, and phenomics. Particularly with the integration of big data analytics and artificial intelligence, GS offers more precise and efficient genetic predictions. These technologies facilitate the creation of tailored breeding programs that can adapt to specific environmental conditions and production requirements. This interdisciplinary cooperation can enable GS to be more effective in improving selection efficiency and breeding progress. As a result, as the cost of genotyping decreases, GS becomes increasingly accessible to a broader range of producers, thus democratizing genetic improvement and fostering greater innovation across the industry. In the long term, the widespread adoption of GS is anticipated to drive significant economic benefits by improving herd productivity, optimizing feed efficiency, and reducing mortality rates, all of which contribute to higher profitability. Additionally, GS aligns with global sustainability goals by facilitating the breeding of livestock with reduced environmental footprints, thus contributing to lower greenhouse gas emissions and promoting environmentally responsible agricultural practices. By utilizing and developing GS, it is promising to find the best model and configuration for various traits, which will strengthen the efficiency and accuracy of selection, accelerate the genetic progress, and provide a foundation for breeding selection, purification, and expansion of sheep and goats.

## Figures and Tables

**Table 1:** Key search terms.

Main Search Term Classification	Specific Search Term Examples
Basic concepts of GS	genomic selection and reference population/marker density/LD/heritability/...
GS method	BLUP/GBLUPssGBLUP/SNPBLUP/Bayes/...
Application of GS in specific species	GS in sheep/goat
GS in meat traits	weight/meat quality/growth rate/fat/muscle conformation/... of sheep/goat
GS in fiber traits	wool/wool weight/fiber diameter/staple fiber strength/... of sheep/goat
GS in dairy traits	milk yield/fat content/somatic cell score/... of sheep/ goat
GS in reproductive traits	lambing number/litter size/... of sheep/goat
Other	breeding and GS/phenotypic information/genetic relationship

**Table 2:** Main influencing factors of GS prediction accuracy.

Factor	Impact Direction	Specific Effects
Size of the reference population	↑	Larger population size leads to higher accuracy.
Reference population gender ratio	↕	Reasonable increase leads to higher accuracy; unreasonable decrease leads to lower accuracy.
Marker density	↑	Higher density leads to higher accuracy.
Linkage disequilibrium level	↑	Higher linkage disequilibrium leads to higher accuracy.
Generation interval between populations	↓	Shorter generation interval leads to higher accuracy.
Heritability of target trait	↑	Higher heritability leads to higher accuracy.
Method and model for GEBV estimation	↕	The most suitable method and model lead to the highest accuracy.

Note: The direction of the impact (increase or decrease) is with color coding (green for increase, red for decrease, yellow for conditional impact).

**Table 3:** Research progress of GS in multi-breed and diverse methods for sheep and goats.

Species	Breeds	Traits	Methods	Accuracy of Prediction	Reference
Goat	French dairy goat	milk production traits, udder shape, and somatic score	ssGBLUP, WssGBLUP	The prediction accuracy of ssGBLUP was higher than that of WssGBLUP except for milk yield.	[47]
	Alpine dairy goat and Saanen dairy goat	milk, protein, and fat yields; protein and fat percentages	GBLUP	The accuracy of GEBV estimation by multiple breeds was significantly higher than that by single breeds.	[32]
	Inner Mongolia Cashmere goat	villus yield, villus length, and villus fine traits	ABLUP, GBLUP, and ssGBLUP	The prediction accuracy of different models followed the trend of ABLUP < GBLUP < ssGBLUP.	[38]
	Alpine dairy goat and Saanen dairy goat	milk production traits, somatic cell score, and some udder-type traits	GBLUP	Adding females to the reference population of males improved accuracy by 5 to 9%.	[42]
	Alpine dairy goat and Saanen dairy goat	udder traits	ssGBLUP	The prediction accuracy using two breeds was higher than that using a single breed.	[11]
	Hybrid dairy goats of British Togenberg goat, Alpine dairy goat, and Saanen dairy goat	milk yield, lactation duration, and other lactation traits	SNPBLUP, ssGBLUP	ssGBLUP was more accurate in this population.	[43]
	French dairy goat	protein content in the milk	ssGBLUP, WssGBLUP	The accuracy of three WssGBLUP was higher than that of the unweighted ssGBLUP.	[45]
	Brazilian Saanen dairy goat	milk production and composition traits	GBLUP, Bayes Cπ, and Bayesian LASSO	GBLUP had better selection performance and lower cost in this reference population.	[48]
	Alpine and Saanen dairy goat	protein and fat content in milk	ssGBLUP	The accuracy of GEBV had been increased compared to traditional selection.	[32]
	Spanish Assaf, Churra	milk coagulation properties and cheese yield-related traits	BLUP, SNPBLUP, and ssGBLUP	The accuracy of GEBV had been increased compared to traditional selection.	[49]
Sheep	Soay Sheep	body weight, length of front and rear legs, coat color, patterns, and so on	GBLUP, Bayes R, Bayes A, Bayes B, and Bayes L	The prediction accuracy of all methods was similar and had high accuracy and strong correlation with each other.	[28]
	New Zealand sheep and Norwegian white sheep	birth weight, weaning weight, carcass weight, and fat	ssGBLUP	The prediction accuracy using two breeds was higher than that using a single breed.	[30]
	Australian sheep (Merino sheep, etc.)	wool weight, fiber diameter, and staple fiber strength	GBLUP, BayesA	The prediction accuracy of GBLUP was slightly higher than that of Bayes A.	[21]
	Australian sheep	the quantity of wool	GBLUP	The GEBV for the quantity of wool was high.	[24]
	Romney, Coperworth, and Pollendale sheep	greasy fleece weight at 12 months	GBLUP	The accuracy of genomic prediction improved to 0.26.	[34]
	Merino sheep and crossbreed Merino sheep	wool quality, yield, and turbid degree	GBLUP, Bayes R	The prediction accuracy of GBLUP and Bayes R was similar in this population.	[53]
	Australian sheep	meat quality and wool production	GBLUP, Bayes RC	The prediction accuracy of Bayes RC was higher than that of GBLUP.	[36]
	French Lacaune dairy sheep	14 milk traits such as milk yield, fat content, and somatic cell score	BLUP, Bayes Cπ, partial least squares (PLS), and sparse PLS	There are minor differences among genomic approaches.	[41]
	Sarda sheep	milk fatty acid content	BLUP, GBLUP, and ssGBLUP	The accuracy of prediction was higher in the older group.	[46]
	Australian Merino sheep	reproductive traits	GBLUP, Bayes R	The accuracy of genomic prediction had been increased in populations with distant genetic relationships, and the two models had similar predictive performance.	[54]

## Data Availability

Data sharing is not applicable to this article, as no datasets were generated or analyzed during the current study.

## List of Abbreviations

Abbreviations	The full name of abbreviations
GS	genomic selection
EBV	estimating breeding value
MAS	marker-assisted selection
SNP	single nucleotide polymorphism
QTL	quantitative trait locus
GEBV	Genomic Estimated Breeding Value
LD	linkage disequilibrium
BLUP	Best Linear Unbiased Prediction
RRBLUP	Ridge Regression Best Linear Unbiased Prediction
GBLUP	Genomic Best Linear Unbiased Prediction
ssGBLUP	single-step Genomic Best Linear Unbiased Prediction
pseudo-BLUP	pseudo-Best Linear Unbiased Prediction
pseudo-ssGBLUP	pseudo-Genomic Best Linear Unbiased Prediction
AGG	Annual Genetic Gain
PLS	partial least squares
WssGBLUP	Weighted single-step Genomic Best Linear Unbiased Prediction
